# Long-term safety and effectiveness of velaglucerase alfa in Gaucher disease: 6-year interim analysis of a post-marketing surveillance in Japan

**DOI:** 10.1186/s13023-021-02119-2

**Published:** 2021-12-04

**Authors:** Rieko Sagara, Masahide Ishigaki, Manami Otsuka, Kei Murayama, Hiroyuki Ida, Jovelle Fernandez

**Affiliations:** 1 Japan Medical Office, Takeda Pharmaceutical Company Limited, 2-1-1, Nihonbashi-honcho, Chuo-ku, Tokyo, 103-8668 Japan; 2 Department of Metabolism, Chiba Children’s Hospital, 579-1, Heta-cho Midori-ku, Chiba, 266-0007 Japan; 3 The Jikei University Hospital, 3-19-18 Nishi-shinbashi, Minato-ku, Tokyo, Japan

**Keywords:** Enzyme replacement therapy, Gaucher disease, Glucosylceramidase, Japan, Surveillance, Post-marketing, Safety, Velaglucerase alfa

## Abstract

**Background:**

Gaucher disease (GD) is caused by reduced lysosomal enzyme β-glucocerebrosidase activity. Heterogeneous genotypes and phenotypes have been observed within GD types and across ethnicities. Enzyme replacement therapy is generally recommended for patients with type 1 GD, the least severe form of GD. In Japan, velaglucerase alfa has a broad indication covering type 1, 2 or 3 GD.

**Methods:**

All patients with type 1, 2, or 3 GD administered velaglucerase alfa 60 U/kg every 2 weeks via intravenous infusion after its launch date in Japan in 2014, were enrolled in a non-interventional, observational post-marketing surveillance (PMS). Individual patient data were reported via case report forms (CRFs). Key safety endpoints investigated included the incidence of infusion-related reactions (IRRs), the safety of velaglucerase alfa in patients with types 2 and 3 GD, from patients under one year of age to elderly patients (≥ 65 years of age). Long-term efficacy was also assessed.

**Results:**

In total, 53 patients with GD were registered. CRFs were available for 41 (77.4%) patients at the 6-year interim analysis. Fourteen adverse drug reactions (ADRs) were reported in seven patients. All reported ADRs occurred in patients with type 2 GD. ADRs were reported by 63.6% (7/11) of patients with type 2 GD. Ten ADRs were reported in five patients aged < 4 years. No elderly patients experienced any ADR during the surveillance period. Five ADRs occurring in three (10.0%) patients were classified as IRRs, with one case of vomiting (moderate severity) resulting in treatment discontinuation. Ten serious adverse events were reported in five (16.7%) patients. Three fatal events were considered to be unrelated to treatment with velaglucerase alfa. Platelet counts increased after the administration of velaglucerase alfa and were generally maintained within the normal range over the administration period. Among eleven patients tested for neutralizing anti-velaglucerase alfa antibodies, two (18.2%) were assessed as positive results.

**Conclusion:**

PMS data from patients with types 1–3 GD in Japan indicate that long-term treatment with velaglucerase alfa was well-tolerated and associated with increased platelet counts, which is consistent with observations made in studies outside of Japan.

*Trial registration*: NCT03625882 registered July 2014.

## Background

Gaucher disease (GD) is a rare, autosomal recessive lipid storage disorder caused by mutations in the *GBA1* gene, leading to reduced activity of the lysosomal enzyme β-glucocerebrosidase [1]. As a result, glucocerebroside accumulates in macrophages (which transform into Gaucher cells), and these cells infiltrate the bone marrow, liver, spleen, and brain, among other organs, resulting in splenomegaly, thrombocytopenia, anemia, hepatomegaly, bone issues and neurological abnormalities [1–3].

GD is classified into three broad phenotypes, based on neurological involvement and age of onset: type 1 (non-neuronopathic), type 2 (acute neuronopathic), and type 3 (chronic neuronopathic) [2, 4, 5]. Type 1 is the most common and mildest form of GD, typically diagnosed during adolescence, whereas type 2 GD is characterized by both systemic involvement and severe neurological impairment presenting during infancy, which is often fatal by 1–3 years of age [1, 2, 4]. Type 3 GD is a highly heterogeneous form that generally presents during early childhood and is characterized by systemic manifestations and varying degrees of neurological involvement [1, 2, 4, 6].

However, the clinical presentation of GD is diverse, characterized by a continuum of severity that results from more than 400 different underlying genetic mutations, highlighting ethnic differences in both genotype and phenotype [2, 4, 7–14]. The thrombocytopenia common to patients with GD is believed to result from defects in hemostatic processes as well as from immune-mediated destruction [15, 16]. Consequently, platelet count tests are routinely employed to monitor the efficacy of therapies for patients with GD [16–18].

Long-term enzyme replacement therapy (ERT) is the current standard of care for patients with GD [1, 2]. ERTs have limited efficacy in treating the neurological symptoms of GD because the infused enzyme molecules are too large to cross the blood–brain barrier. However, ERT can still be beneficial for patients with type 2 or 3 GD, by improving visceral and hematological manifestations and quality of life [6, 19]. Long-term ERT with velaglucerase alfa has demonstrated an ability to stabilize the symptoms of GD and slow disease progression, improving platelet counts, hemoglobin levels, plasma biomarkers and liver and spleen volume in patients with type 1 disease [20, 21].

There are currently two available therapies for the treatment of GD in Japan; imiglucerase and velaglucerase alfa which were approved in 1998 and 2014, respectively. Both enzymes are indicated for the treatment of all types of GD at a recommended dosage of 60 units/kg/dose every 2 weeks [22, 23]. Of these two products, only velaglucerase alfa is produced in a human cell line and is identical to naturally occurring human glucocerebrosidase (GBA); imiglucerase differs by one amino acid and has a different glycosylation pattern [2, 24, 25].

Velaglucerase alfa has demonstrated efficacy and tolerability in several studies in adult and pediatric patients with Type 1 GD, including both treatment-naïve patients [20, 21, 26–29] and those previously treated with imiglucerase [30–33]. However, the vast majority of patients enrolled in these studies were from non-Asian countries, and no Japanese patients were included. Therefore, such findings may not be generalizable to Japanese patients with GD. Type 1 GD accounts for 43% of the total GD population in Japan, but, comparatively, type 1 accounts for 94% of GD diagnoses in western countries [34]. Further, Japanese patients with GD have been reported to have more severe symptoms compared with non-Japanese patients, and novel mutations have been identified in Japanese patients with GD [12]. Studies by Schwartz et al. demonstrated that velaglucerase alfa is well-tolerated and associated with stable improvements in hemoglobin concentration, platelet counts, and liver and spleen volumes over 24 months in five Japanese patients with type 1 (n = 3) or 3 (n = 2) GD, previously treated with imiglucerase [35]. Hence, velaglucerase alfa was approved for the treatment of type 1, 2 and 3 GD in Japan in July 2014 [23]. Comparatively, velaglucerase alfa was only approved for the treatment of type 1 GD in the US and EU [36, 37]. To date, data concerning the long-term safety and efficacy of velaglucerase alfa in the real-world setting are lacking. Therefore, this post-marketing surveillance (PMS) (ClinicalTrials.gov identifier: NCT03625882) was undertaken in Japan to collect data on the long-term safety of velaglucerase alfa for the treatment of GD and to investigate the changes in platelet counts after treatment with velaglucerase alfa. Here, we report interim results after the first 6 years of this 8-year surveillance.

## Results

**Fig. 1:**
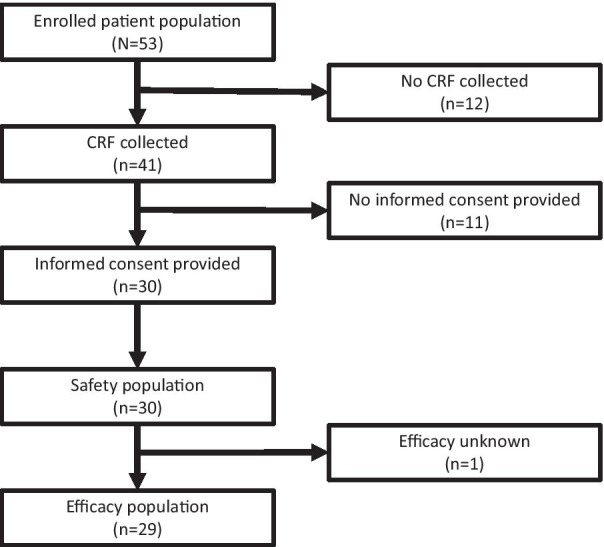
Patient disposition. Demonstration of the patient progression through the study, from enrollment to allocation within a study group. The number of patients included within each stage are shown. Abbreviation: CRF, case report form

Between July 2014 and February 2020, 53 patients with GD were registered as part of the PMS. Case report forms (CRFs) were available for 41 (77.4%) patients at the 6-year interim analysis (Fig. 1). CRFs had not yet been collected during the surveillance period for 12 (22.6%) patients. An additional eleven (20.8%) patients did not provide written informed consent for their data to be included in a scientific publication. Patient demographics for 30 (56.6%) patients who had CRFs and consented to their data being used in a scientific publication were consistent with the expected presentation for each type of GD (Table 1). Ten (33.3%) patients were < 18 years of age, six (20.0%) of whom were < 4 years of age, and 23 (76.7%) patients had switched from prior ERT, all of whom had received imiglucerase. The median duration of exposure to velaglucerase alfa was 3 years.

**Table 1:** Patient demographics

	Type 1 GD (N = 8)	Type 2 GD (N = 11)	Type 3 GD (N = 11)	Total (N = 30)
Sex, n (%)				
Male	5 (62.5)	6 (54.5)	6 (54.5)	17 (56.7)
Female	3 (37.5)	5 (45.5)	5 (45.5)	13 (43.3)
Age, years, mean (SD)	53.5 (12.6)	3.4 (3.8)	20.1 (4.7)	22.9 (21.4)
Treatment status, n (%)				
Naïve	3 (37.5)	3 (27.3)	1 (9.1)	7 (23.3)
Experienced	5 (62.5)	8 (72.7)	10 (90.9)	23 (76.7)

Basic demographics data including sex, age and treatment status are displayed for patients with Type 1, 2 and 3 GD

*GD* Gaucher disease, *SD* standard deviation

### Safety

In total, 14 adverse drug reactions (ADRs) were reported in seven patients (Table 2). All ADRs were reported in patients with type 2 GD; no ADRs were reported by patients with type 1 or type 3 GD. ADRs were reported by 63.6% of patients with type 2 GD and 10 ADRs were reported in five patients (83.3%) aged < 4 years, who experienced two ADRs each. Three ADRs were reported by two treatment-naïve patients (28.6%). These ADRs occurred during both the early and late phases of treatment. Seven ADRs (50%) occurred within 6 months of treatment, three between 6 months and a year, and 2 over a year after the initial administration of velaglucerase alfa (for the remaining two ADRs the onset days were unknown). Patients reported repeat incidences of vomiting, hyperchlorhydria or pyrexia within 100 days after the initiation of treatment with velaglucerase alfa. There was no clear association between the time a patient had been on ERT treatment and the onset of ADRs. No elderly patients experienced any ADR during the surveillance period.

**Table 2:** ADRs reported for Japanese patients with GD according to MedDRA version 22.1

	Total (N = 30)	Type 2 GD (N = 11)	Age < 4 years (N = 6)	Treatment-naïve (N = 7)
Patients with ADRs, n (%)	7 (23.3)	7 (63.6)	5 (83.3)	2 (28.6)
ADRs, n	14	14	10	3
Patients with serious ADRs, n (%)	5 (16.7)	1 (9.1)	1 (16.7)	1 (14.3)
Serious ADRs, n	10	2	2	2
Type of ADR				
Antibody test positive	2	2	1	0
Pyrexia	2	2	2	1
Vomiting	2	2	1	0
Aspartate aminotransferase increased	1	1	1	0
Erythema	1	1	1	0
Gastric hypomotility	1	1	1	1
Hyperchlorhydria	1	1	1	1
Myoclonus	1	1	0	0
Rash	1	1	0	0
Swelling of eyelid	1	1	1	0
Tachycardia	1	1	1	0

The different forms of ADR are presented for patients, following treatment with velaglucerase alfa, showing the results for all patients as well as the results for those with type 2 GD, those < 4 years of age and those who were treatment naïve

*ADR* adverse drug reaction, *GD* Gaucher disease, *MedDRA* Medical Dictionary for Regulatory Activities

Among the seven patients reporting ADRs, five ADRs occurring in three patients (10.0%) were classified as infusion-related reactions (IRRs) (Table 3). The five IRRs comprised one event of: pyrexia, vomiting, erythema, tachycardia and swelling of the eyelid and all were mild or moderate in severity. The case of vomiting was classified as a serious IRR of moderate severity, resulting in treatment discontinuation. All IRRs resolved.

**Table 3:** IRRs reported for Japanese patients with GD

	Total	Severity	Action taken	Outcome
Patients with IRRs, n (%)	3 (10)			
IRRs, n	5			
Erythema	1	Moderate	Dose reduced	Resolved
Pyrexia	1	Mild	No change	Resolved
Swelling of eyelid	1	Mild	No change	Resolved
Tachycardia	1	Moderate	Dose reduced	Resolved
Vomiting	1	Moderate	Drug withdrawn	Resolved
Patients with serious IRRs, n (%)	1 (3)			
Vomiting	1		Drug withdrawn	Resolved

All infusion-related reactions following treatment with velaglucerase alfa are presented, as well as data on the severity, action taken and outcome

*IRR* infusion-related reactions

Ten serious adverse events were reported in five patients (16.7%); each of the five patients experienced two events. Recorded according to MedDRA, one event each of acute myeloid leukemia, bile duct stone, bipolar disorder, cardiac failure congestive, cellulitis, cholelithiasis, duodenal ulcer, myelodysplastic syndrome, sepsis and vomiting were reported.

Three fatal events were reported (as per MedDRA; cardiac failure congestive, myelodysplastic syndrome and acute myeloid leukemia), but all were considered to be unrelated to treatment with velaglucerase alfa. One patient discontinued treatment due to vomiting, despite recovering after medicative and non-medicative therapy.

### Efficacy

**Fig. 2:**
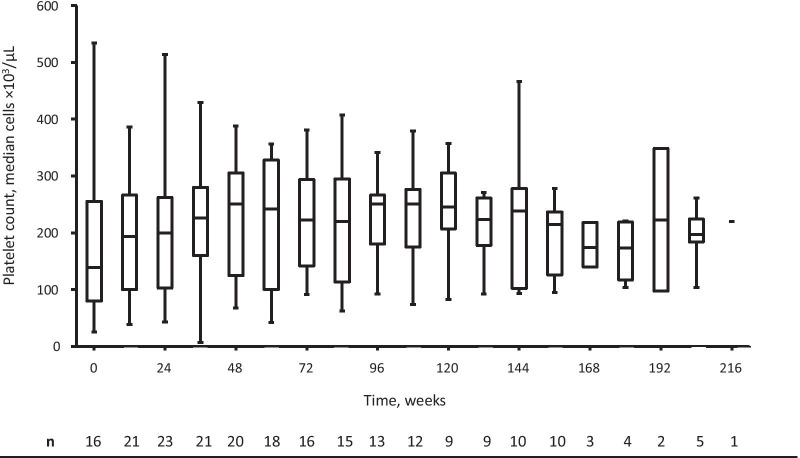
Platelet counts for Japanese patients with GD treated with velaglucerase alfa. Box and whisker chart showing the change in platelet count in the patients with GD following treatment with velaglucerase alfa over time. *GD* Gaucher disease

In total, 29 patients were included in the efficacy analysis, however variable numbers of patients were recorded for each time point. Platelet counts increased after the administration of velaglucerase alfa and were generally maintained within the normal range over the administration period. The median (Q1, Q3) platelet count was 138.5 (79.5, 255.5) × 10^3^/μL (n = 16) at baseline and increased by 18.0 (-2.0, 70.0), 31.5 (1.0, 131.5), and 29.5 (-18.0, 84.0) × 10^3^/μL at weeks 24 (n = 15), 48 (n = 12), and 96 (n = 8), respectively (Fig. 2).

### Anti-velaglucerase alfa antibodies

Among eleven patients tested for neutralizing anti-velaglucerase alfa antibodies, two (18.2%) were assessed as positive results.

## Discussion

In the 6 years since velaglucerase alfa was launched for the treatment of type 1, 2, or 3 GD in Japan, treatment was found to be well-tolerated among patients with type 1 or 3 GD, with ADRs limited to patients with type 2 GD. Furthermore, IRRs were only reported by 10% of patients, all of which were classified as being mild or moderate in severity, only resulting in treatment discontinuation for a single patient. Velaglucerase alfa was also efficacious, increasing mean platelet counts. A favorable long-term safety and tolerability profile has previously been observed for velaglucerase alfa after 4 years of follow-up in phase 3 trials of patients with type 1 GD outside Japan, with therapeutic goals for thrombocytopenia, splenomegaly, anemia, hepatomegaly and bone mineral density being met in most patients [20, 21, 28]. However, the indication for velaglucerase alfa outside of Japan has also been limited to treating patients with type 1 GD [36, 37]. Furthermore, Japanese patients tend to have a more severe type 1 GD phenotype than Jewish patients with type 1 GD [38]. Published data on the use of velaglucerase alfa to treat GD (type 1 and type 3) in Japan for a 24-month trial are limited to five patients who switched from imiglucerase [35].

Before this surveillance, there were no data on the use of velaglucerase alfa to treat ERT-naïve patients with GD in Japan. Furthermore, there are limited data on the safety and efficacy of velaglucerase alfa in patients with Type 2 and 3 GD globally, so understanding the clinical profile of velaglucerase alfa in these patient groups offers additional insight into the management options for these conditions.

The proportion of patients reporting IRRs in this surveillance is consistent with an earlier study in more than 200 patients with type 1 GD, which reported that 13.3% of patients experienced an IRR with velaglucerase alfa [31]. However, despite IRRs generally being reported within the first three injections, IRRs have been reported to occur several years after initiating treatment with ERT, so patients receiving velaglucerase alfa may need to continue to be monitored for IRRs throughout their treatment, although discontinuation due to an IRR is uncommon [28, 39].

Producing velaglucerase alfa using human cell lines and gene activation technology may assist in limiting the risk of IRRs by theoretically lowering the risk of immunogenicity and the formation of anti-velaglucerase alfa antibodies [21]. A pooled analysis of patients with GD treated with velaglucerase alfa in clinical trials between 2004 and 2015, also found that only four (1.4%) patients developed anti-velaglucerase alfa antibodies, including two patients with neutralizing antibodies, without apparent pharmacodynamic impact or effect on clinical response [40]. In this surveillance, details of the two patients who returned positive tests for anti-velaglucerase alfa antibodies were not sufficiently described to determine any impact on clinical benefit. However, data continue to be collected relating to the incidence and effects of developing antibodies to velaglucerase alfa in Japanese patients. Due to the small number of patients enrolled in the surveillance, it is difficult to explain the cause of the adverse reactions in type 2 GD patients, however, the more severe disease experienced by type 2 patients compared with type 1 or 3 GD patients may be a factor. Only 11 antibody tests were performed during the surveillance at the physicians' discretion; one patient was found positive for neutralizing anti-velaglucerase alfa antibodies at Month 6 and another at Month 12. As such, no clear relationship could be found between antibody positivity and disease type.

The surveillance data presented here are limited due to their interim nature as well as the low patient numbers. Of the 41 patients whose CRFs were collected, data from 30 patients were included within this analysis after clinical data from patients who did not provide their informed consent were omitted. In addition, no post hoc analysis was initially conducted on pediatric patients: no pediatric patients were enrolled in the clinical trial during development. The current post-marketing surveillance study mainly evaluated safety. The authors will conduct a post hoc analysis to further explore this issue. However, these findings still provide valuable real-world evidence of the safety and efficacy of velaglucerase alfa in patients with GD. Because the study included patients who were treated with other ERTs before treatment with velaglucerase alfa, the median platelet counts at baseline were not very low, a further limitation of this surveillance. Further analysis is needed to show how much velaglucerase alfa improves platelet counts in drug naïve patients or patients switched from other ERTs.

## Conclusion

In conclusion, PMS data from patients with types 1, 2 and 3 GD in Japan indicate that long-term treatment with velaglucerase alfa was well-tolerated and associated with increased platelet counts, which is consistent with observations made in studies outside of Japan. These interim surveillance findings also provide insights into clinical outcomes for patients with type 2 and 3 GD, supporting the use of velaglucerase alfa in these populations. Completion of this PMS is awaited to provide a fuller evaluation of the real-world safety and efficacy of velaglucerase alfa in GD.

## Methods

### Surveillance design

As a mandatory condition for approval, all patients treated with velaglucerase alfa in Japan were enrolled in this PMS. This protocol was designed, and surveillance was conducted, in accordance with the Japanese Good Post-marketing Study Practice guidelines [41] as a non-interventional, observational interim assessment, evaluating safety and efficacy of velaglucerase alfa in patients with Type 1, 2 or 3 GD in Japan within the approved condition including the indication, dosage and precautions. The approved indication for velaglucerase alfa is “Improvement of various symptoms of Gaucher disease (anemia, thrombocytopenia, hepatosplenomegaly, and bone symptoms)”. All medicines were used under the terms of the authorized indication and dose schedule, if the physicians thought it necessary. This protocol was reviewed by the Institutional committee at each medical institution, however, according to the good post-marketing study practice (GPSP) in Japan, this is not a requirement for PMS. Additionally, all patients included in this analysis provided written informed consent for their data to be included in this publication. In the case of minors, written informed consent was provided by a legal representative or guardian.

Patients of any age or sex with a confirmed diagnosis of GD type 1, 2, or 3 who were either treatment-naïve or previously treated with an ERT other than velaglucerase alfa were eligible to participate, provided they were administered velaglucerase alfa 60 U/kg every 2 weeks via intravenous infusion in Japan after its launch date in 2014 and registered to participate in the surveillance [23]. Diagnosis was confirmed by physicians by testing for defective GBA activity or identifying specific mutations in the *GBA* gene. All patients were followed continuously until end of survey, death, or the withdrawal of velaglucerase alfa for any reason.

### Data collection

Individual patient data were reported from July 2014, via CRFs derived from patient medical records. These included safety and efficacy data recorded as part of routine clinical practice when treating patients with GD using velaglucerase alfa. Baseline characteristics, including patient demographics, type of GD, clinical symptoms, comorbidities, prior treatments, date of velaglucerase alfa initiation and dose were also recorded.

Safety information collected included adverse events, defined as any undetectable sign, symptom or disease (e.g., abnormal laboratory findings) occurring after administering velaglucerase alfa, regardless of whether or not it was considered by a physician to be related to velaglucerase alfa administration. Serious adverse events or serious drug reactions were defined as being life-threatening at the time of occurrence, medically significant, requiring patient hospitalization (or prolongation of existing hospitalization), resulting in persistent disability/incapacity, a congenital anomaly or birth defect or death. Hypersensitivity reactions were defined as events of drug allergy, angioedema, anaphylactic reaction or shock, anaphylactic reaction, anaphylaxis, anaphylaxis treatment, or drug reaction with eosinophilia and systemic symptoms. Each ADR was summarized by preferred term (MedDRA/J version 22.1).

Additional safety data collected included the presence of anti-velaglucerase alfa neutralizing antibodies (NAB), and any IRRs, defined as any reaction that occurred within 24 h of administering velaglucerase alfa. Investigations into the presence of NABs were performed at the discretion of the physician under normal clinical practice with samples confirmed as positive for anti-velaglucerase alfa antibodies using a NAB assay.

Key safety endpoints investigated included the incidence of IRRs, the safety of velaglucerase alfa in treatment-naïve patients, patients with types 2 and 3 GD, patients under the age of 4 years and elderly patients (age ≥ 65 years). For the efficacy population, the key efficacy data captured in the CFR was the platelet count which was measured approximately every 12 weeks at the physician’s discretion, as per standard clinical practice.

### Statistical analysis

Data cut-off for this interim analysis was 25 February 2020. Data were summarized for patients overall and according to previous treatment status and GD type. Continuous variables were summarized using descriptive statistics and categorical variables by the number and percentage of patients in each category. No formal hypothesis testing was undertaken. Statistical analyses were performed using SAS version 9.4.

## Data Availability

The datasets, including the redacted surveillance protocol, redacted statistical analysis plan, and individual participant's data supporting the results reported in this article, will be made available within three months from initial request to researchers who provide a methodologically sound proposal. The data will be provided after its de-identification, in compliance with applicable privacy laws, data protection and requirements for consent and anonymization.

## Abbreviations

ADR: Adverse drug reaction
ERT: Enzyme replacement therapy
GBA: Glucocerebrosidase
GD: Gaucher disease
IRR: Infusion-related reaction
NAB: Neutralizing antibody
PMS: Post-marketing surveillance
